# Unusual Case of a Torted Mesenteric Fibroid

**DOI:** 10.1155/2018/8342127

**Published:** 2018-06-07

**Authors:** Rawan Bajis, Gregg Eloundou

**Affiliations:** ^1^King Edward Memorial Hospital, Australia; ^2^Joondalup Health Campus, Australia

## Abstract

Extrauterine leiomyomas are very rare and present a clinical and diagnostic challenge due to their unusual growth patterns and behaviours. A 47-year-old woman was transferred to our tertiary specialist obstetrics and gynaecology hospital with acute abdominal pain and a palpable abdominal mass. She was taken immediately to theatre with the presumptive diagnosis of an ovarian torsion. Intraoperatively, a large necrotic mass originating from the mesentery and attachments to the bowel at the ileocaecal junction was noted. When converted to laparotomy due to limited access and poor visualisation, the uterus, ovaries, and tubes were found to be normal. A right partial hemicolectomy was performed with the assistance of the colorectal surgeon due to suspicion of bowel malignancy. Histology revealed a benign infarcted leiomyoma with adhesions to the adjacent ileum. The diagnosis of a primary torted mesenteric fibroid was made.

## 1. Introduction

Leiomyomas (fibroids) are histologically distorted smooth muscle tumours, which are thought to arise from the genitourinary tract and are almost always benign [1]. They are very common, occurring in up to 65% of women and are incidentally found in approximately 80% of all uterine specimens at hysterectomy [2, 3]. They are estrogen and progesterone dependent which might explain their prevalence in the female reproductive years as well as their regression following menopause [2]. They represent the most common gynaecological tumour and cause severe clinical symptoms in up to 30% of women [4]. Women with uterine fibroids often present with a variety of symptoms depending on their anatomical location and size. These could range from abnormal vaginal bleeding, pelvic pain, and pressure symptoms against neighbouring organs, to subfertility [5–7]. Rarely, fibroids can occur and grow in unusual locations outside the uterus and in doing so present a clinical and diagnostic challenge [8]. Due to their unusual growth patterns and behaviours, patients present with a wide variety of symptoms and on occasions can be acutely unwell, mimicking other gynaecological or nongynaecological conditions and in some cases mimicking other malignancies. As a result, the diagnosis of most extrauterine fibroids is often only made intraoperatively, either during diagnostic laparoscopy for acute presentations of pain or during surgery for management of other previously diagnosed abdominal-pelvic masses. They can also be diagnosed incidentally on various imaging modalities such as ultrasound, CT, or MRI performed for unrelated conditions [8].

Little is known about these rare extrauterine growths. In fact, it is often only after histological assessment of the resected specimen that a diagnosis of a leiomyoma is made. It has been postulated that these atypical leiomyomas develop secondary to cell spillage and seeding during morcellation at hysterectomy or myomectomy [9, 10]. Rarely, as in this case, have extrauterine fibroids been reported in the absence of previous fibroid surgery and hence why the physiopathology of primary solitary mesenteric leiomyoma development in a “virgin” abdomen is not clearly recognised or understood.

We present a case of a 46-year-old woman with a torted mesenteric fibroid; her puzzling presentation, intraoperative findings, and suggested theories behind the possible development are also discussed.

## 2. Case Presentation

A 47-year-old para 2 presented to the general hospital emergency department with a 3-day history of severe abdominal pain, nausea, and vomiting. This was her first hospital presentation with abdominal pain. Her past medical history included two previous caesarean sections but no other abdominal surgeries. A month prior to this presentation, she had visited her primary healthcare provider complaining of a distended abdomen. An ultrasound performed at the time had reported a large pedunculated uterine fibroid measuring 126x104x108mm. The left ovary was normal but the right ovary was not visualised. Unfortunately, this information was not readily available to the treating team at the time of her acute presentation.

A FAST scan (focused assessment with sonography for trauma) done in the general hospital had reported a moderate amount of free fluid in the right iliac fossa and a large pelvic mass. The presumed diagnosis was an ovarian cyst torsion and formed the premise for transfer to our tertiary specialist obstetrics and gynaecology hospital.

On arrival to our specialist hospital, the patient was haemodynamically stable and afebrile. On palpation of her abdomen, a large, firm, mobile, and tender mass was noted, extending to her umbilicus. She had a negative pregnancy test. Besides a raised CRP of 77ml/L, the rest of her blood profile was normal.

**Figure 1:**
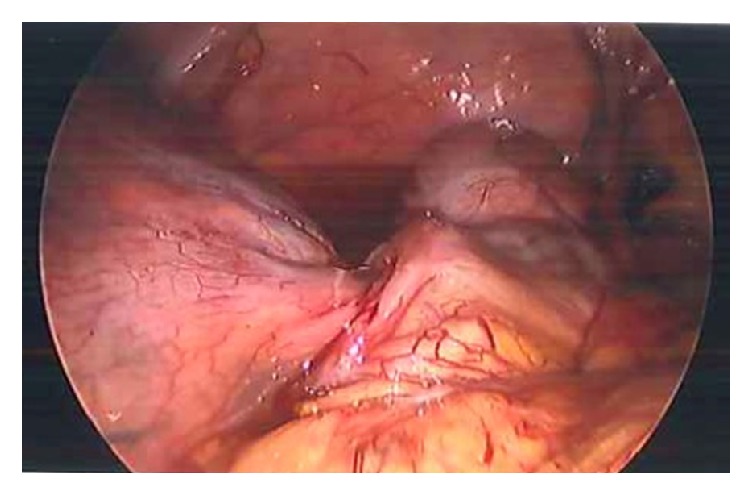
Laparoscopic view of fibroid (right) and its attachment to the bowel (left). Area of torsion noted in between.

**Figure 2:**
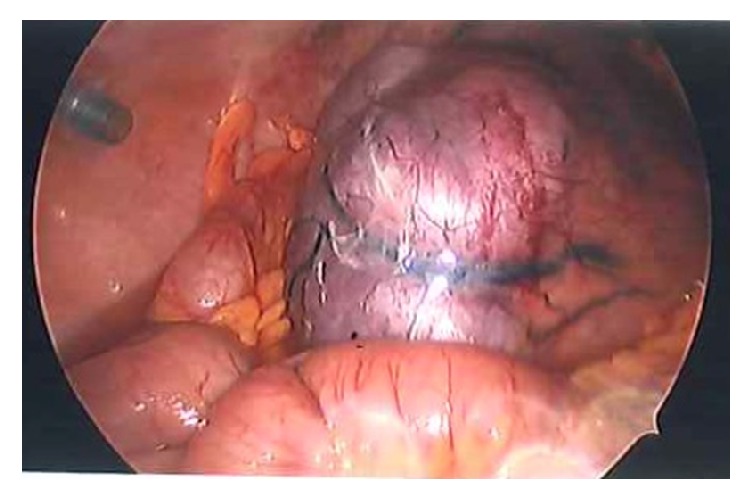
Laparoscopic view of infarcted fibroid.

Given the acute nature of her presentation and the ultrasound findings, she was transferred to theatre for laparoscopic management of a torted ovarian cyst. At laparoscopy, performed through Palmers point entry due to the large mass extending to the umbilicus, a large 25cm multilobulated necrotic mass occupying the entire lower abdomen and pelvis was found. The mass was mobile with attachments to the bowel at the right iliac fossa. Due to limited laparoscopic accessibility, it was impossible to fully explore the rest of the pelvic organs safely. Laparoscopy was converted to a laparotomy and a transverse lower abdominal incision was made for a safer inspection. A large necrotic mass originating from the mesentery of the ileocaecal junction was noted (Figure 1). Its borders were confluent with the walls of the terminal ileum at the ileocaecal junction, which in itself had become necrotic having axially rotated under the weight of the growth (Figure 2). This raised the suspicion of a bowel malignancy. The appendix could not be clearly visualised. The uterus, ovaries, and tubes appeared normal. Specifically, the uterus showed no areas of detachment. The on-call colorectal surgeon was contacted to assist with the remainder of the surgery due to extensive bowel involvement. The right colon and terminal ileum were mobilized and the mesentery was dissected to the level of the origin of the ileocolic vessels. A right partial hemicolectomy was performed with a primary ileocolic anastomosis and the mesenteric defect closed. A frozen section was considered, however, due to the large nature of the necrotic mass and extensive bowel involvement with adjacent necrosis, the entire specimen was resected* en bloc*. Postoperatively, she had a five-day hospital stay complicated by an ileus requiring nasogastric tube placement. She made a full recovery and was discharged home with outpatient clinic follow-up.

On macroscopic histopathology, a large mass attached to the serosal surface of the ileocaecal junction with a 10mm vascular stalk was reported. Microscopically, a circumscribed mass composed of interlacing bundles of smooth muscle with large areas of necrosis and infarction was noted. Adhesions made up of fibrofatty tissue, between the mass and the caecum, were present. The smooth muscle cells stained positive for desmin and actin and showed a low Ki-67 staining pattern. Features of a benign leiomyoma with extensive infarction were confirmed. Adhesions between the leiomyoma and the adjacent ileum and caecum were extensively present, supporting the diagnosis of a torted extrauterine mesenteric fibroid.

## 3. Discussion

Extrauterine fibroids are extremely rare and poorly understood. They develop outside the uterus and arise as proliferation of smooth muscle cells with behaviours and histological characteristics identical to their uterine leiomyoma counterparts [8]. Their diagnosis is complicated by their nonspecific clinical presentation. A history of a hysterectomy or fibroid surgery with morcellation is often an associated finding and may be suggestive of the diagnosis and aetiology [11]. Extrauterine fibroids usually present with acute pain and complications secondary to compression affects as a consequence of their location [8]. Common presentations include bowel obstruction, haemorrhage, and, as in this case, infarction resulting in an acute abdomen and severe pain. Their appearance may mimic that of malignancy dictating the need for a wide resection.

Unusual extrauterine fibroid growth patterns described in the literature to date include peritoneal leiomyomatosis, intravenous leiomyomas, benign metastasizing leiomyomas, retroperitoneal leiomyomas, and parasitic leiomyomas. Although various theories and hypothesis exist regarding the mechanism behind these manifestations, no established conclusive pathophysiology has been made. It is important however to consider the heterogeneous theories that do exist regarding the bizarre manifestations of these extrauterine leiomyomas, before considering the diagnosis of our case.

Disseminated peritoneal leiomyomatosis, first described by Taubert et al. in 1965, is a rare condition characterized by innumerable diffuse vascular peritoneal nodules that grow along submesothelial tissues in the abdominopelvic peritoneum. It is thought that these multiple growths arise from smooth muscle metaplasia of submesothelial mesenchymal cells, the embryological origins of the peritoneum that line the female reproductive tract [12]. These are almost always benign but sarcomatous transformations have been described [13]. Hormonal factors have also been implicated in their pathogenesis as case reports of these extrauterine masses have been documented in association with pregnancy, hormone secreting ovarian tumours, and the combined contraceptive pill [14, 15].

Another rare extrauterine pathology exists in the form of Intravenous leiomyomas. These manifest as intramural proliferation of leiomyomas that occur within intrauterine and systemic veins [16]. They are believed to grow within vessels of the myometrium and parametrium with reported cases of extensions to the inferior vena cava and the heart [17]. These are almost always associated with previous uterine fibroid surgery.

Benign metastasizing leiomyomas, as first described in 1939, manifest as nodules of well-differentiated benign leiomyomatous tissue located at distal sites to the uterus, mimicking metastatic disease [18]. Most commonly, these benign metastatic lesions are located in the lung and have been noted up to 20 years after surgery for known uterine fibroids [1, 19]. These are hormone receptor positive tumours and have been reported to self-resolve in postmenopausal patients. It is on this premise that hormone modulators may provide the rationale behind treatment of symptomatic individuals [20, 21]. Lymphovascular embolization of uterine leiomyomatous tissue at distant sites is the most commonly recognised theory [21].

As first described in the literature by Kelly et al. in 1909, parasitic leiomyomas are a rare type of migratory leiomyoma. It was first hypothesised that pedunculated subserosal leiomyomas could adhere to surrounding structures, detach from the uterus, and develop axillary blood supplies, thus explaining the diagnoses of leiomyomas at sites distal to a myomatous uterus [22]. Although rare, they have been reported to occur more frequently in the broad ligament, pelvic peritoneum, and omentum [1]. A second hypothesis exists whereby cell spillage and seeding take place during morcellation at time of surgical resection of the uterine fibroid or at hysterectomy. Kho et al. reported a case series of 12 parasitic myomas in 2009, noting several leiomyomas adherent to nearby bowel mesentery, appendix, anterior abdominal wall, and rectovaginal septum [10]. In all but two cases, women had previously undergone abdominal surgery for known fibroid or endometriosis. This case series supported the previously hypothesised theory that prior surgery, specifically hysterectomy or myomectomy with morcellation, is a potential risk factor for the development of extrauterine fibroids, specifically iatrogenic parasitic myomas. As such, the Food and Drug Administration of USA has recently advised against the continued use of power morcellators in treatment of uterine fibroids [23]. Although a review regarding the use of morcellation therapy was initially sparked due to the increased reports of iatrogenic abdominal and peritoneal seeding of uterine sarcoma, the increased prevalence of extrauterine fibroids has also played a part in discouraging its use [24]. Parasitic myomas remain rare but may become more frequently reported with increased use of laparoscopic surgery in gynaecology. Therefore, it should be considered as a differential in any female presenting with acute abdominal pain or with an abdominal-pelvic mass with known history of fibroid surgery [11].

Our case presented a diagnostic dilemma and left us puzzled as to the possible pathophysiological explanation. The most probable mechanism for her diagnosis would be that of a parasitic myoma. This is reinforced by the fact that she had had two previous caesarean sections and an ultrasound scan a month prior to her presentation reporting the presence of a subserosal pedunculated fibroid. However, caesarean sections have not yet been postulated as a potential source for seeding. Also, at the time of surgery, the uterus appeared normal with no area of detachment noted. Given the degree of dense adhesion to bowel and mesentery, and the short length of time that had elapsed between her scan and presentation, it is possible that the migration theory could not be the sole explanation. This case could therefore represent a solitary primary mesenteric fibroid.

In the literature to date, there is little evidence of primary mesenteric fibroids in an unmorcellated abdomen. In a case series published by Dan et al. in 2012 of two patients who required right hemicolectomies for extrauterine fibroids, one patient had no prior history of abdominal surgery, whereas the other had a history of a laparoscopic myomectomy just eleven days prior [25]. Whether there is predilection for the right abdomen is unclear; however, in this case series and the one we report here, all three patients had right hemicolectomies for leiomyoma's morbidly adherent to the mesentery of the right-sided colon.

What is even rarer is the presence of a torted extrauterine leiomyoma. In our case, the fibroid had rotated along a stalk morbidly adherent to underlying mesentery and bowel. There are few reported cases in the literature describing torted parasitic fibroids in the absence of having had previous abdominal surgeries [26–28]. Similar to our case, however, Yeh et al. in 1999 published a case report of a woman who presented with a torted fibroid of the broad ligament with no uterine wall attachment and no prior history of abdominal surgery [28]. That too could be considered a primary extrauterine fibroid similar to the one we are presenting. Whatever the definition, the mechanism remains a mystery.

It is important to consider the possibility of an extrauterine fibroid as a differential diagnosis in any female presenting with an intra-abdominal and pelvic mass, especially with a history of previous surgery for fibroids. It is also important to recognise the need for prompt exploratory surgery in any premenopausal patient presenting with acute abdominal pain and the clinical suspicion of torsion.

Our case highlights the complexity of the diagnosis of extrauterine fibroids and adds to the body of literature describing and reporting extrauterine fibroids. As they remain rare and grossly misunderstood, it is prudent to continue reporting cases in order to create a database which could be used to formulate a better understanding of the clinical presentation, diagnosis, and management.
